# (2*RS*)-2-(2,4-Difluoro­phen­yl)-1-[(4-iodo­benz­yl)(meth­yl)amino]-3-(1*H*-1,2,4-tri­azol-1-yl)propan-2-ol

**DOI:** 10.1107/S160053681203139X

**Published:** 2012-07-14

**Authors:** Hui-Ping Xiong, Shou-Hong Gao, Chun-Tong Li, Zhi-Jun Wu

**Affiliations:** a School of Mathematics and Physics, Shanghai University of Electric Power, Shanghai 200090, People’s Republic of China; b Department of Pharmacy, Changzheng Hospital, Second Military Medical University, Shanghai 200003, People’s Republic of China

## Abstract

In the title compound (common name: iodiconazole), C_19_H_19_F_2_IN_4_O, there is an intra­molecular O—H⋯N hydrogen bond and mol­ecules are linked by weak inter­actions only, namely C—H⋯N, C—H⋯O and C—H⋯F hydrogen bonds, and π-electron ring–π-electron ring inter­actions between the triazole rings with centroid–centroid distances of 3.725 (3) Å.

## Related literature

For the pharmacological activity of azole compounds, see Fromtling (1988 ▶); Gallagher *et al.* (2003 ▶). For a liquid chromatography-tandem mass spectrometry (LC-MS/MS) assay for determination of trace amounts of iodiconazole in human plasma, see Gao *et al.* (2009 ▶). For an ultra-fast LC method for the determination of iodiconazole in microdialysis samples and its application in the calibration of laboratory-made linear probes, see Sun *et al.* (2010 ▶). For the high-performance liquid chromatographic (HPLC) determination of iodiconazole in rat plasma, see Wen *et al.* (2007 ▶). For the synthesis of iodiconazole, see Sheng *et al.* (2002 ▶); Zhang *et al.* (2001 ▶). For classification of the hydrogen bonds, see Gilli & Gilli (2009 ▶).
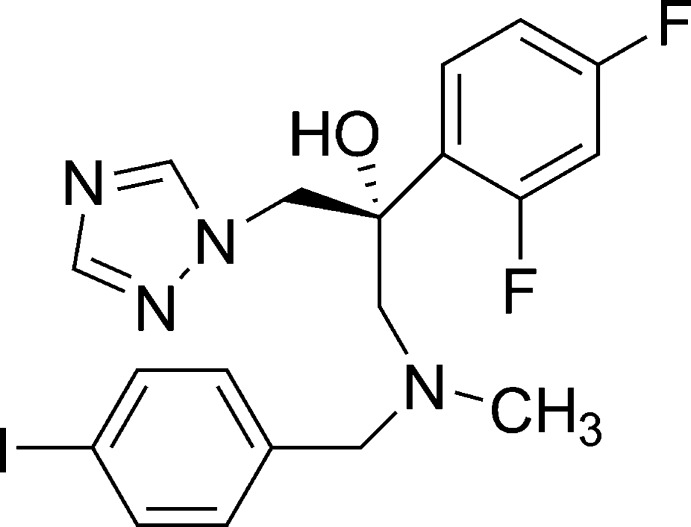


## Experimental

### 

#### Crystal data

C_19_H_19_F_2_IN_4_O
*M* *_r_* = 484.28
Monoclinic, 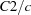

*a* = 34.398 (14) Å
*b* = 5.812 (2) Å
*c* = 21.619 (9) Å
β = 114.895 (5)°
*V* = 3921 (3) Å^3^
*Z* = 8
Mo *K*α radiation
μ = 1.67 mm^−1^
*T* = 293 K
0.30 × 0.25 × 0.25 mm

#### Data collection

Bruker SMART APEX diffractometer
Absorption correction: multi-scan (*SADABS*; Sheldrick, 1996 ▶) *T*_min_ = 0.635, *T*_max_ = 0.681
8473 measured reflections
3929 independent reflections
3441 reflections with *I* > 2σ(*I*)
*R*_int_ = 0.035

#### Refinement

*R*[*F* ^2^ > 2σ(*F* ^2^)] = 0.039
*wR*(*F* ^2^) = 0.103
*S* = 1.07
3929 reflections
249 parameters
1 restraint
H atoms treated by a mixture of independent and constrained refinement
Δρ_max_ = 0.66 e Å^−3^
Δρ_min_ = −0.89 e Å^−3^

### 

Data collection: *SMART* (Bruker, 1997 ▶); cell refinement: *SAINT* (Bruker, 1997 ▶); data reduction: *SAINT*; program(s) used to solve structure: *SHELXS97* (Sheldrick, 2008 ▶); program(s) used to refine structure: *SHELXL97* (Sheldrick, 2008 ▶); molecular graphics: *SHELXTL* (Sheldrick, 2008 ▶); software used to prepare material for publication: *SHELXTL*.

## Supplementary Material

Crystal structure: contains datablock(s) I, global. DOI: 10.1107/S160053681203139X/fb2257sup1.cif

Structure factors: contains datablock(s) I. DOI: 10.1107/S160053681203139X/fb2257Isup2.hkl

Supplementary material file. DOI: 10.1107/S160053681203139X/fb2257Isup3.cml

Additional supplementary materials:  crystallographic information; 3D view; checkCIF report

## Figures and Tables

**Table 1:** Hydrogen-bond geometry (Å, °)

*D*—H⋯*A*	*D*—H	H⋯*A*	*D*⋯*A*	*D*—H⋯*A*
C9—H9*B*⋯N2	0.97	2.60	3.045 (4)	108
C9—H9*B*⋯N4	0.97	2.42	3.191 (4)	136
C12—H12*A*⋯O1	0.93	2.39	2.759 (4)	103
C17—H17*A*⋯F1	0.97	2.43	3.061 (4)	122
O1—H1⋯N1	0.81 (2)	1.97 (3)	2.651 (4)	141 (4)

**Fig. 1.**
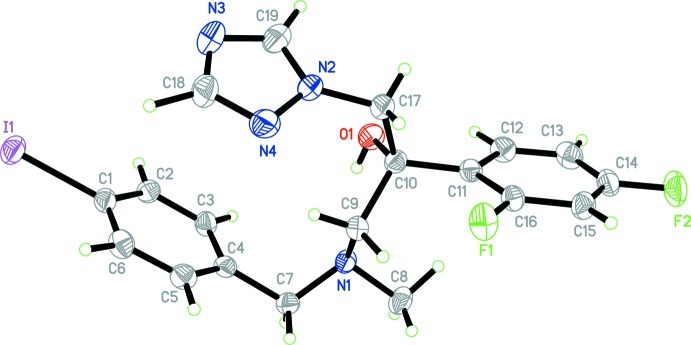
The title molecule with the atom-labelling scheme. The displacement ellipsoids are drawn at the 30% probability level. The H atoms are shown as small spheres of arbitrary radius.

## supplementary crystallographic information

### Comment

Azole antifungal drugs play chief role in the treatment of fungal infections. Azole drugs are advantageous because they undergo stable metabolism and can be applied either *per os* or by injection. They are efficient for internal and external fungal infections (Gallagher *et al.*, 2003), too. In order to obtain new compounds with more potent activity, less toxicity and a broader antifungal spectrum, several azole compounds have been synthesized (Sheng *et al.*, 2002; Zhang *et al.*, 2001). Herein we report the crystal structure determination of the title compound which belongs to the same chemical class.

There is an intramolecular O1—H1···N1 hydrogen bond of moderate strength in the structure. (Table 1; For classification of the hydrogen bonds, see Gilli & Gilli, 2009). The molecules are linked by weak C—H···N, C—H···O and C—H···F hydrogen bonds (Table 1). Moreover, there are π-electron ring—π-electron ring interactions between the triazole rings with the centroid distances of 3.725 (3) Å with the symmetry code of the second ring is -*x*, *y*, 3/2-*z*.

### Experimental

The title compound was prepared according to the procedure described by Sheng *et al.* (2002): To a stirred mixture of 1-[2-(2,4-difluorophenyl)-2,3-epoxypropyl]-1*H*-1,2,4-triazole methanesulphonate (3 g, 0.009 mol), anhydrous CH_3_OH (20 ml) and NaOH (0.4 g), 4-iodo-N-methyl-benzylamine (4.46 g, 0.022 mol) was added. The mixture was heated at 50–60 C° for 6 h. The reaction was monitored by thin-layer chromatography (TLC). The resulting mixture was kept at room temperature for 12 h. After filtration, the filtrate was evaporated under reduced pressure. Water (50 ml) was added to the residue and it was extracted with ethyl acetate (3 × 100 ml). The extract was washed with saturated NaCl solution (50 ml × 3), dried over anhydrous Na_2_SO_4_ and evaporated under vacuum. The residue was purified by column chromatography on silica gel (petroleum ether: EtOAc 1: 1 *v*/*v*) to afford iodiconazole. Single crystals (colourless prisms) were grown by slow evaporation of a solution of the title compound in petroleum ether/acetone (1:1, *v*/*v*) at room temperature.

### Refinement

All the hydrogens were discernible in the difference electron density map. Despite of it the hydrogens attached to the C atoms were treated in the riding atom formalism: C_aryl_—H=0.93 , C_methyl_—H=0.96, C_methylene_—H=0.97 Å. *U*_iso_(H)=1.2*U*_eq_(C_aryl/methylene_), *U*_iso_(H)=1.5*U*_eq_(C_methyl_). The positional parameters of the hydroxyl hydrogen H1 were refined applying the distance restraint O1-H1 distance equal to 0.82 (2) Å. *U*_iso_(H1)=1.5*U*_eq_(O1).

### Crystal data

**Table:**

C_19_H_19_F_2_IN_4_O	*F*(000) = 1920
*M**_r_* = 484.28	*D*_x_ = 1.641 Mg m^−^^3^
Monoclinic, *C*2/*c*	Mo *K*α radiation, λ = 0.71073 Å
Hall symbol: -C 2yc	Cell parameters from 935 reflections
*a* = 34.398 (14) Å	θ = 2.4–27.3°
*b* = 5.812 (2) Å	µ = 1.67 mm^−^^1^
*c* = 21.619 (9) Å	*T* = 293 K
β = 114.895 (5)°	Prism, colourless
*V* = 3921 (3) Å^3^	0.30 × 0.25 × 0.25 mm
*Z* = 8	

### Data collection

**Table:**

Bruker SMART APEX diffractometer	3929 independent reflections
Radiation source: fine-focus sealed tube	3441 reflections with *I* > 2σ(*I*)
Graphite monochromator	*R*_int_ = 0.035
ω scans	θ_max_ = 26.3°, θ_min_ = 1.9°
Absorption correction: multi-scan (*SADABS*; Sheldrick, 1996)	*h* = −42→37
*T*_min_ = 0.635, *T*_max_ = 0.681	*k* = −7→6
8473 measured reflections	*l* = −20→26

### Refinement

**Table:**

Refinement on *F*^2^	Secondary atom site location: difference Fourier map
Least-squares matrix: full	Hydrogen site location: difference Fourier map
*R*[*F*^2^ > 2σ(*F*^2^)] = 0.039	H atoms treated by a mixture of independent and constrained refinement
*wR*(*F*^2^) = 0.103	*w* = 1/[σ^2^(*F*_o_^2^) + (0.0625*P*)^2^ + 1.2617*P*] where *P* = (*F*_o_^2^ + 2*F*_c_^2^)/3
*S* = 1.07	(Δ/σ)_max_ < 0.001
3929 reflections	Δρ_max_ = 0.66 e Å^−^^3^
249 parameters	Δρ_min_ = −0.89 e Å^−^^3^
1 restraint	Extinction correction: *SHELXL97* (Sheldrick, 2008), Fc^*^=kFc[1+0.001xFc^2^λ^3^/sin(2θ)]^-1/4^
72 constraints	Extinction coefficient: 0.0042 (2)
Primary atom site location: structure-invariant direct methods	

### Special details

**Table:**

Geometry. All e.s.d.'s (except the e.s.d. in the dihedral angle between two l.s. planes) are estimated using the full covariance matrix. The cell e.s.d.'s are taken into account individually in the estimation of e.s.d.'s in distances, angles and torsion angles; correlations between e.s.d.'s in cell parameters are only used when they are defined by crystal symmetry. An approximate (isotropic) treatment of cell e.s.d.'s is used for estimating e.s.d.'s involving l.s. planes.
Refinement. Refinement of F^2^ against ALL reflections. The weighted R-factor wR and goodness of fit S are based on F^2^, conventional R-factors R are based on F, with F set to zero for negative F^2^. The threshold expression of F^2^ > 2σ (F^2^) is used only for calculating R-factors(gt) etc. and is not relevant to the choice of reflections for refinement. R-factors based on F^2^ are statistically about twice as large as those based on F, and R- factors based on ALL data will be even larger.

### Fractional atomic coordinates and isotropic or equivalent isotropic displacement parameters (Å^2^)

**Table:**

	*x*	*y*	*z*	*U*_iso_*/*U*_eq_	
I1	0.039478 (7)	0.71329 (5)	1.084656 (12)	0.05727 (14)	
O1	0.14979 (9)	0.4892 (4)	0.86484 (13)	0.0526 (6)	
H1	0.1635 (13)	0.562 (7)	0.8990 (16)	0.079*	
F1	0.12653 (7)	1.0421 (3)	0.72386 (10)	0.0579 (5)	
F2	0.21734 (8)	0.6966 (5)	0.64091 (13)	0.0730 (7)	
N1	0.18407 (7)	0.8775 (4)	0.92893 (12)	0.0381 (5)	
N2	0.06131 (9)	0.6265 (5)	0.81152 (15)	0.0512 (7)	
N3	0.02469 (12)	0.4885 (7)	0.8643 (2)	0.0786 (11)	
N4	0.04556 (10)	0.8147 (6)	0.8307 (2)	0.0631 (9)	
C1	0.08547 (10)	0.8148 (6)	1.04983 (16)	0.0428 (7)	
C2	0.11877 (10)	0.6647 (5)	1.05788 (17)	0.0432 (7)	
H2A	0.1202	0.5207	1.0775	0.052*	
C3	0.14984 (11)	0.7318 (5)	1.03632 (17)	0.0423 (7)	
H3A	0.1722	0.6319	1.0421	0.051*	
C4	0.14813 (9)	0.9441 (5)	1.00639 (15)	0.0388 (6)	
C5	0.11418 (11)	1.0879 (5)	0.99801 (16)	0.0451 (7)	
H5A	0.1122	1.2298	0.9769	0.054*	
C6	0.08326 (10)	1.0275 (6)	1.01995 (17)	0.0486 (7)	
H6A	0.0612	1.1288	1.0147	0.058*	
C7	0.18278 (10)	1.0181 (5)	0.98510 (15)	0.0432 (7)	
H7A	0.1782	1.1778	0.9708	0.052*	
H7B	0.2103	1.0078	1.0242	0.052*	
C8	0.22209 (10)	0.9452 (7)	0.91844 (18)	0.0532 (8)	
H8A	0.2239	0.8528	0.8829	0.080*	
H8B	0.2474	0.9226	0.9599	0.080*	
H8C	0.2198	1.1045	0.9056	0.080*	
C9	0.14435 (9)	0.9017 (5)	0.86661 (15)	0.0383 (6)	
H9A	0.1464	1.0359	0.8415	0.046*	
H9B	0.1205	0.9248	0.8787	0.046*	
C10	0.13590 (9)	0.6840 (5)	0.82054 (16)	0.0378 (6)	
C11	0.15886 (9)	0.6898 (5)	0.77369 (15)	0.0375 (6)	
C12	0.18560 (10)	0.5119 (5)	0.77230 (17)	0.0471 (7)	
H12A	0.1903	0.3885	0.8020	0.056*	
C13	0.20542 (11)	0.5125 (6)	0.72800 (19)	0.0535 (8)	
H13A	0.2232	0.3917	0.7280	0.064*	
C14	0.19842 (11)	0.6929 (6)	0.68468 (18)	0.0490 (8)	
C15	0.17194 (11)	0.8736 (6)	0.68202 (16)	0.0473 (7)	
H15A	0.1671	0.9949	0.6516	0.057*	
C16	0.15292 (9)	0.8656 (5)	0.72667 (15)	0.0392 (6)	
C17	0.08801 (11)	0.6515 (6)	0.77506 (18)	0.0499 (8)	
H17A	0.0779	0.7828	0.7448	0.060*	
H17B	0.0845	0.5160	0.7470	0.060*	
C18	0.02440 (14)	0.7188 (9)	0.8626 (3)	0.0735 (13)	
H18A	0.0101	0.8059	0.8827	0.088*	
C19	0.04840 (13)	0.4356 (8)	0.8317 (2)	0.0675 (11)	
H19A	0.0551	0.2866	0.8239	0.081*	

### Atomic displacement parameters (Å^2^)

**Table:**

	*U*^11^	*U*^22^	*U*^33^	*U*^12^	*U*^13^	*U*^23^
I1	0.04130 (16)	0.0808 (2)	0.05347 (18)	−0.01084 (10)	0.02357 (12)	−0.00551 (11)
O1	0.0739 (16)	0.0366 (11)	0.0525 (14)	−0.0017 (11)	0.0318 (13)	0.0096 (10)
F1	0.0711 (12)	0.0534 (11)	0.0545 (11)	0.0266 (10)	0.0318 (10)	0.0207 (9)
F2	0.0674 (15)	0.1079 (19)	0.0620 (14)	−0.0039 (13)	0.0450 (13)	−0.0128 (13)
N1	0.0346 (12)	0.0465 (13)	0.0348 (12)	−0.0034 (10)	0.0161 (10)	0.0018 (10)
N2	0.0443 (15)	0.0601 (16)	0.0555 (17)	−0.0121 (13)	0.0270 (14)	−0.0102 (14)
N3	0.069 (2)	0.095 (3)	0.092 (3)	−0.020 (2)	0.054 (2)	−0.009 (2)
N4	0.0486 (17)	0.067 (2)	0.078 (2)	−0.0083 (14)	0.0309 (17)	−0.0192 (17)
C1	0.0370 (15)	0.0553 (18)	0.0365 (15)	−0.0055 (13)	0.0159 (13)	−0.0072 (13)
C2	0.0497 (17)	0.0382 (15)	0.0451 (17)	−0.0023 (12)	0.0232 (15)	0.0025 (12)
C3	0.0460 (17)	0.0410 (15)	0.0448 (17)	0.0051 (12)	0.0241 (15)	0.0024 (13)
C4	0.0432 (15)	0.0389 (15)	0.0341 (14)	−0.0047 (12)	0.0160 (12)	−0.0046 (11)
C5	0.0547 (17)	0.0373 (15)	0.0426 (17)	0.0024 (13)	0.0198 (14)	0.0048 (13)
C6	0.0446 (16)	0.0522 (18)	0.0471 (18)	0.0099 (14)	0.0174 (14)	0.0009 (14)
C7	0.0460 (15)	0.0450 (16)	0.0392 (16)	−0.0108 (13)	0.0184 (13)	−0.0038 (13)
C8	0.0389 (15)	0.074 (2)	0.0492 (19)	−0.0104 (15)	0.0214 (14)	0.0001 (17)
C9	0.0384 (14)	0.0405 (15)	0.0380 (15)	0.0016 (12)	0.0179 (12)	0.0011 (12)
C10	0.0399 (15)	0.0367 (14)	0.0394 (16)	−0.0013 (11)	0.0192 (13)	0.0020 (12)
C11	0.0396 (15)	0.0373 (14)	0.0350 (15)	−0.0014 (11)	0.0152 (13)	0.0002 (11)
C12	0.0521 (17)	0.0379 (15)	0.0497 (18)	0.0068 (13)	0.0200 (15)	0.0032 (13)
C13	0.0469 (17)	0.0555 (19)	0.060 (2)	0.0086 (14)	0.0242 (16)	−0.0091 (16)
C14	0.0409 (17)	0.069 (2)	0.0398 (17)	−0.0059 (15)	0.0197 (14)	−0.0122 (15)
C15	0.0487 (17)	0.0569 (18)	0.0345 (16)	−0.0043 (15)	0.0157 (14)	0.0051 (14)
C16	0.0407 (15)	0.0408 (15)	0.0366 (15)	0.0042 (12)	0.0167 (13)	0.0015 (12)
C17	0.0450 (17)	0.064 (2)	0.0450 (18)	−0.0134 (15)	0.0234 (15)	−0.0101 (15)
C18	0.049 (2)	0.104 (4)	0.081 (3)	−0.012 (2)	0.040 (2)	−0.025 (3)
C19	0.061 (2)	0.068 (2)	0.087 (3)	−0.0139 (19)	0.044 (2)	−0.007 (2)

### Geometric parameters (Å, º)

**Table:**

I1—C1	2.103 (3)	C6—H6A	0.9300
O1—C10	1.429 (4)	C7—H7A	0.9700
O1—H1	0.810 (19)	C7—H7B	0.9700
F1—C16	1.354 (3)	C8—H8A	0.9600
F2—C14	1.356 (4)	C8—H8B	0.9600
N1—C9	1.467 (4)	C8—H8C	0.9600
N1—C8	1.472 (4)	C9—C10	1.560 (4)
N1—C7	1.480 (4)	C9—H9A	0.9700
N2—C19	1.335 (5)	C9—H9B	0.9700
N2—N4	1.360 (4)	C10—C11	1.525 (4)
N2—C17	1.448 (4)	C10—C17	1.534 (4)
N3—C19	1.320 (5)	C11—C12	1.393 (4)
N3—C18	1.339 (6)	C11—C16	1.394 (4)
N4—C18	1.319 (6)	C12—C13	1.390 (5)
C1—C6	1.382 (5)	C12—H12A	0.9300
C1—C2	1.391 (5)	C13—C14	1.358 (5)
C2—C3	1.390 (5)	C13—H13A	0.9300
C2—H2A	0.9300	C14—C15	1.375 (5)
C3—C4	1.383 (4)	C15—C16	1.376 (4)
C3—H3A	0.9300	C15—H15A	0.9300
C4—C5	1.385 (4)	C17—H17A	0.9700
C4—C7	1.510 (4)	C17—H17B	0.9700
C5—C6	1.380 (5)	C18—H18A	0.9300
C5—H5A	0.9300	C19—H19A	0.9300
			
C10—O1—H1	96 (3)	C10—C9—H9A	109.4
C9—N1—C8	112.2 (2)	N1—C9—H9B	109.4
C9—N1—C7	111.3 (2)	C10—C9—H9B	109.4
C8—N1—C7	108.3 (2)	H9A—C9—H9B	108.0
C19—N2—N4	109.8 (3)	O1—C10—C11	110.0 (2)
C19—N2—C17	129.5 (3)	O1—C10—C17	107.3 (3)
N4—N2—C17	120.7 (3)	C11—C10—C17	107.1 (2)
C19—N3—C18	102.5 (4)	O1—C10—C9	107.2 (2)
C18—N4—N2	101.4 (3)	C11—C10—C9	113.4 (2)
C6—C1—C2	120.0 (3)	C17—C10—C9	111.7 (3)
C6—C1—I1	121.1 (2)	C12—C11—C16	115.1 (3)
C2—C1—I1	118.9 (2)	C12—C11—C10	122.0 (3)
C3—C2—C1	119.4 (3)	C16—C11—C10	122.9 (3)
C3—C2—H2A	120.3	C13—C12—C11	122.2 (3)
C1—C2—H2A	120.3	C13—C12—H12A	118.9
C4—C3—C2	121.3 (3)	C11—C12—H12A	118.9
C4—C3—H3A	119.3	C14—C13—C12	118.8 (3)
C2—C3—H3A	119.3	C14—C13—H13A	120.6
C3—C4—C5	117.9 (3)	C12—C13—H13A	120.6
C3—C4—C7	120.9 (3)	F2—C14—C13	119.7 (3)
C5—C4—C7	121.2 (3)	F2—C14—C15	117.6 (3)
C6—C5—C4	122.0 (3)	C13—C14—C15	122.7 (3)
C6—C5—H5A	119.0	C14—C15—C16	116.5 (3)
C4—C5—H5A	119.0	C14—C15—H15A	121.7
C5—C6—C1	119.3 (3)	C16—C15—H15A	121.7
C5—C6—H6A	120.3	F1—C16—C15	116.8 (3)
C1—C6—H6A	120.3	F1—C16—C11	118.5 (3)
N1—C7—C4	113.2 (2)	C15—C16—C11	124.7 (3)
N1—C7—H7A	108.9	N2—C17—C10	114.8 (3)
C4—C7—H7A	108.9	N2—C17—H17A	108.6
N1—C7—H7B	108.9	C10—C17—H17A	108.6
C4—C7—H7B	108.9	N2—C17—H17B	108.6
H7A—C7—H7B	107.8	C10—C17—H17B	108.6
N1—C8—H8A	109.5	H17A—C17—H17B	107.5
N1—C8—H8B	109.5	N4—C18—N3	116.0 (4)
H8A—C8—H8B	109.5	N4—C18—H18A	122.0
N1—C8—H8C	109.5	N3—C18—H18A	122.0
H8A—C8—H8C	109.5	N3—C19—N2	110.3 (4)
H8B—C8—H8C	109.5	N3—C19—H19A	124.9
N1—C9—C10	111.1 (2)	N2—C19—H19A	124.9
N1—C9—H9A	109.4		

### Hydrogen-bond geometry (Å, º)

**Table:**

*D*—H···*A*	*D*—H	H···*A*	*D*···*A*	*D*—H···*A*
C9—H9*B*···N2	0.97	2.60	3.045 (4)	108
C9—H9*B*···N4	0.97	2.42	3.191 (4)	136
C12—H12*A*···O1	0.93	2.39	2.759 (4)	103
C17—H17*A*···F1	0.97	2.43	3.061 (4)	122
O1—H1···N1	0.81 (2)	1.97 (3)	2.651 (4)	141 (4)

## References

[bb1] Bruker (1997). *SMART* and *SAINT* Bruker AXS Inc., Madison, Wisconsin, USA.

[bb2] Fromtling, R. A. (1988). *Clin. Microbiol. Rev.* **1**, 187–217. 10.1128/cmr.1.2.187 PMC358042 3069196

[bb3] Gallagher, J. G., Dodds Ashley, E. S., Drew, R. H. & Perfect, J. R. (2003). *Expert Opin. Pharmacother.* **4**, 147–164. 10.1517/14656566.4.2.147 12562305

[bb4] Gao, S. H., Tao, X., Sun, L. N., Sheng, C. Q., Zhang, W. N., Yun, Y. L., Li, J. X., Miao, H. J. & Chen, W. S. (2009). *J. Chromatogr. B*, **877**, 382–386. 10.1016/j.jchromb.2008.12.034 19124284

[bb5] Gilli, G. & Gilli, P. (2009). *The Nature of the Hydrogen Bond. Outline of a Comprehensive Hydrogen Bond Theory*, p. 61. International Union of Crystallography Book Series. Oxford, New York: Oxford University Press.

[bb6] Sheldrick, G. M. (1996). *SADABS* University of Göttingen, Germany.

[bb7] Sheldrick, G. M. (2008). *Acta Cryst.* A**64**, 112–122. 10.1107/S0108767307043930 18156677

[bb8] Sheng, C. Q., Zhang, W. N., Ji, H. T., Zhou, Y. J., Song, Y. L., Zhou, J., Lu, J. G. & Yang, S. (2002). *J. Chin. Pharm. Sci.* **11**, 5–10.

[bb9] Sun, N., Wen, J., Lu, G., Hong, Z. Y., Fan, G. R., Wu, Y. T., Sheng, C. Q. & Zhang, W. N. (2010). *J. Pharm. Biomed. Anal.* **51**, 248–251. 10.1016/j.jpba.2009.07.016 19695826

[bb10] Wen, J., Fan, G. R., Hong, Z. Y., Chai, Y. F., Yin, Y. T., Sheng, C. Q. & Zhang, W. N. (2007). *J. Pharm. Biomed. Anal.* **50**, 580–586. 10.1016/j.jpba.2006.07.043 16950589

[bb11] Zhang, W. N., Ji, H. T., Zhou, Y. J., Lu, J. G., Zhou, J., Liu, X. L., Zhang, L. & Zhu, J. (2001). Chin. Patent No. CN1292378A.

